# Using the Negative Soil Adjustment Factor of Soil Adjusted Vegetation Index (SAVI) to Resist Saturation Effects and Estimate Leaf Area Index (LAI) in Dense Vegetation Areas

**DOI:** 10.3390/s21062115

**Published:** 2021-03-17

**Authors:** Zhijun Zhen, Shengbo Chen, Tiangang Yin, Eric Chavanon, Nicolas Lauret, Jordan Guilleux, Michael Henke, Wenhan Qin, Lisai Cao, Jian Li, Peng Lu, Jean-Philippe Gastellu-Etchegorry

**Affiliations:** 1 College of Geoexploration Science and Technology, Jilin University, Changchun 130026, China; zhijun.zhen@univ-tlse3.fr (Z.Z.); qinwenhan@jlu.edu.cn (W.Q.); cls@jlu.edu.cn (L.C.); orinatureli@jlu.edu.cn (J.L.); lupeng@jlu.edu.cn (P.L.); 2 CESBIO—UPS, CNES, CNRS, IRD, Université de Toulouse, CEDEX 9, 31401 Toulouse, France; eric.chavanon@cesbio.cnes.fr (E.C.); nicolas.lauret@univ-tlse3.fr (N.L.); guilleuxj@cesbio.cnes.fr (J.G.); 3 Earth System Science Interdisciplinary Center, University of Maryland, College Park, MD 20740-3823, USA; tiangang.yin.85@gmail.com; 4 Leibniz Institute of Plant Genetics and Crop Plant Research (IPK Gatersleben), OT Gatersleben, Corrensstr 3, Stadt Seeland, D-06466 Gatersleben, Germany; mhenke@uni-goettingen.de

**Keywords:** dense forest, google earth engine (GEE), leaf area index (LAI), remote sensing (RS), soil adjustment factor, soil adjusted vegetation index (SAVI)

## Abstract

Saturation effects limit the application of vegetation indices (VIs) in dense vegetation areas. The possibility to mitigate them by adopting a negative soil adjustment factor *X* is addressed. Two leaf area index (LAI) data sets are analyzed using the Google Earth Engine (GEE) for validation. The first one is derived from observations of MODerate resolution Imaging Spectroradiometer (MODIS) from 16 April 2013, to 21 October 2020, in the Apiacás area. Its corresponding VIs are calculated from a combination of Sentinel-2 and Landsat-8 surface reflectance products. The second one is a global LAI dataset with VIs calculated from Landsat-5 surface reflectance products. A linear regression model is applied to both datasets to evaluate four VIs that are commonly used to estimate LAI: normalized difference vegetation index (NDVI), soil adjusted vegetation index (SAVI), transformed SAVI (TSAVI), and enhanced vegetation index (EVI). The optimal soil adjustment factor of SAVI for LAI estimation is determined using an exhaustive search. The Dickey-Fuller test indicates that the time series of LAI data are stable with a confidence level of 99%. The linear regression results stress significant saturation effects in all VIs. Finally, the exhaustive searching results show that a negative soil adjustment factor of SAVI can mitigate the SAVIs’ saturation in the Apiacás area (i.e., *X* = −0.148 for mean LAI = 5.35), and more generally in areas with large LAI values (e.g., *X* = −0.183 for mean LAI = 6.72). Our study further confirms that the lower boundary of the soil adjustment factor can be negative and that using a negative soil adjustment factor improves the computation of time series of LAI.

## 1. Introduction

As major land cover of the planet, forests have become a key priority in studies of the biodiversity and the carbon cycle of terrestrial ecosystems [1,2]. It is essential to record forest dynamics to understand the terrestrial carbon cycle better and improve forest management practices [3,4]. Leaf area index (LAI), defined as the one-sided green leaf area per unit ground area in broadleaf canopies and one-half the total needle surface area per unit ground area in coniferous canopies, is an essential indicator for describing the canopy structure of forest ecosystems, estimating the primary productivity of the stand, and evaluating forest condition over large areas [5,6].

A number of studies link the LAI and radiometric measurements of vegetation. In particular, Vegetation indices (VIs) are often used to estimate LAI from broad spectral bands [7,8]. Although their analytical expressions differ significantly, the implementations of these indices can be divided roughly into three categories: (1) Intrinsic VIs such as simple ratio (SR) [9] and normalized difference vegetation index (NDVI) [10]. (2) Soil adjusted VIs such as soil adjusted vegetation index (SAVI) [11], transformed SAVI (TSAVI) [12], modified SAVI (MSAVI) [13], modified transformed SAVI (MTSAVI) [14], optimized SAVI (OSAVI) [15], and generalized SAVI (GESAVI) [16]. (3) Atmospheric corrected VIs such as atmospherically resistant vegetation index (ARVI) [17], global environment monitoring index (GEMI) [18], enhanced vegetation index (EVI) [19]. Intrinsic VIs [9,10] are widely used because of their simplicity [8]. They are usually the linear combination of reflectance values in the red and near-infrared (NIR) bands. Based on the intrinsic VIs, soil-adjusted VIs [11,12,13,14,15,16] add a soil adjustment factor and/or soil line parameter (slope and intersection) to mitigate soil noise’s impact on VIs. Atmospheric corrected VIs [17,18,19] add another atmosphere adjustment factor based on soil adjusted indices to mitigate the impact of atmosphere on VIs.

Despite their wide variety and that they are usually designed to correlate with LAI [12], all VIs inevitably suffer from saturation effects [19]. However, for LAI values more than a certain threshold, the derivative of VIs is relative to LAI decreases. This is the so-called saturation effect [12,20]. Saturation is primarily due to the low sensitivity of reflectance in the red band [8,20]. It directly limits the application of VIs in areas of dense vegetation areas. A number of methods have been developed to address saturation effects on NDVI. Ünsalan and Boyer [21] suggested transforming the NDVI by using an inverse tangent function. However, the transformed NDVI does not boost VIs’ sensitivity for vegetation fractions greater than 0.6 [22]. Gitelson [23] and Vaiopoulos et al. [24] further proposed to adjust the NIR and red reflectances relative contributions to NDVI by adding weighting factors to the NIR reflectance term. However, these weighting factors do not account for the influence of the soil noise and alter the dynamic range of the NDVI, resulting in a range of −0.6 to 0.6 [23].

Therefore, in this paper, we addressed the possibility of adjusting the soil adjustment factor to mitigate the saturation effects. Time series of NDVI, SAVI, TSAVI, and EVI calculated from Sentinel-2, Landsat-5, and Landsat-8 surface reflectance products were used to estimate the time series LAI. The latter ones were validated with two LAI data sets, including a time series of MODIS LAI data over the Apiacás area in Brazil and a global LAI dataset. The positive and negative soil adjustment factor performances were then compared based on the correlation coefficients of the linear regression model.

## 2. Data and Methods

### 2.1. Data

Our study uses two data sets: MODIS 4-Day global 500 m LAI product (MCD15A3H V6 level 4, provided by the National Aeronautics and Space Administration Land Processes Distributed Active Archive Center at the U.S. Geological Survey Earth Resources Observation and Science Center which is located outside Sioux Falls, South Dakota, USA) from time-series observation data and the global LAI product global leaf area index from field measurements (GLAIFM) from field measurements. MODIS LAI data were used to study a region, Apiacá, for which VIs were calculated from Sentinel-2 and Landsat-8 surface reflectance. For the global field-measured LAI dataset, the VIs were calculated with Landsat-5 surface reflectance products.

#### 2.1.1. Local MODIS Time Series LAI Data

**Figure 1:**
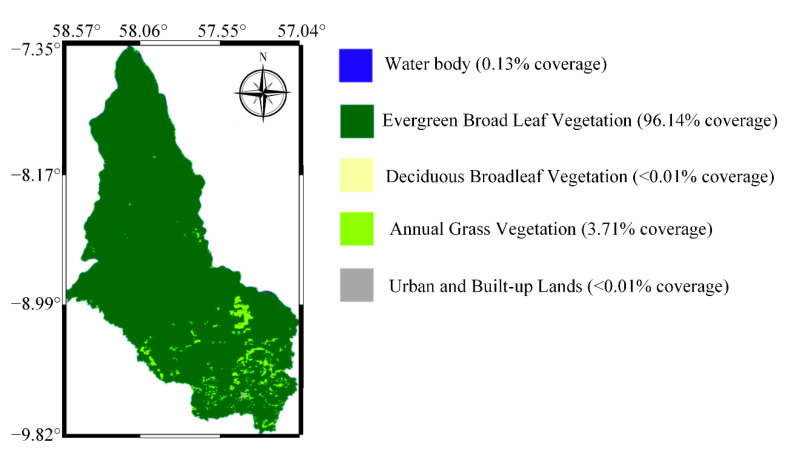
Base map of the study area from the MODIS classification product (MCD12Q1 V6). It has five types of land cover: water body (blue), evergreen broadleaf vegetation (dark green), deciduous broadleaf vegetation (yellow), annual grass vegetation (yellow-green), and urban and built-up lands (dark gray).

Our study includes the entire Apiacás (Latitude: −7.35°–−9.82° N, Longitude: 57.04°–58.57° W) in the northernmost of Mato Grosso, Brazil. It was chosen because of its dense vegetation (Figure 1). It contains part of the 19,582 square kilometers of Juruena National Park, one of the largest conservation units in Brazil. Its elevation is around 200 m [25].

The multispectral satellite datasets and MODIS LAI datasets used in this study were provided and processed by the Google Earth Engine [26,27,28] (GEE, https://earthengine.google.com/, accessed on 11 March 2021). LAI data were from the MODIS 4-Day global 500 m LAI products (MCD15A3H V6 level 4). They were used to validate LAI estimation from VIs calculated from surface multispectral reflectance datasets computed with Sentinel-2 images from 4 February 2019, to 10 November 2020, and Landsat-8 Tier 1 images from 16 April 2013, to 21 October 2020. The use of multiple sources of data helped us to increase the sampling frequency of the time series. Because of the similarity of satellite resolution of Sentinel-2 (10 m) and Landsat-8 (30 m), the VIs differences caused by the two sensors were neglected.

First, the multispectral images were processed. All available Sentinel-2 and Landsat-8 Tier 1 surface reflectance data in GEE before 10 November 2020, were selected and then filtered by two rules: selecting images that cover the study area and an average cloud cover lower than 20%. Then, each remaining image was clipped to ensure that only the study area’s pixels were retained. After that, two boundary masks were used in a preprocessing stage. The first was the cloud mask, and the second was the vegetation mask. GEE provides cloud masks of Sentinel-2 and Landsat-8. Pixels in the cloud mask were removed in the subsequent data processing. The vegetation mask of Sentinel-2 was created through the classification of the NDVI and simple ratio NIR/Green Ratio Vegetation Index (GRVI) [29] images using thresholds indicated in a technical report of Sentinel-2 [30]: pixels with (NDVI > 0.40) or (0.36 ≤ NDVI ≤ 0.40 and GRVI > 2.50) were marked as vegetation pixels. The same rule was implemented to create a Landsat-8 vegetation mask. VIs and reflectance values were calculated pixelwise, and the final VIs and reflectance of each satellite image were derived by aggregating VIs and reflectance calculated per pixel. In the next step, LAI was extracted for each corresponding satellite image.

In this work, we used MODIS leaf area index 4-day global 500 m (MCD15A3H V6, https://doi.org/10.5067/MODIS/MCD12Q1.006, accessed on 11 March 2021) [31]. LAI data were searched for each surface reflectance image within eight days of the acquisition time of the surface reflectance image. If no LAI product was available, LAI data within 16-day intervals was searched instead. If no LAI data was available within 16 days, this particular surface reflectance image was ignored. After getting the LAI data in the time range of satellite images, we extracted pixels within both the study area boundary and reflectance image boundary, and a mean LAI value was derived by aggregating all pixels’ LAI inside these two boundaries. Finally, for each multispectral image, we had a mean value of different VIs, a mean value of reflectance in various bands, and a mean value of LAI.

Depending on the type and location of sensors, the study area could correspond to several remote sensing images on the same day. In that case, mean values of VIs, reflectance, and LAI were calculated using the images acquired on that day. The reason for considering the entire study area rather than pixels as one study object was to smooth out inconsistencies caused by differences in the resolution of the remotely sensed data.

#### 2.1.2. Global Field Measured LAI Data

We used global LAI data from the GLAIFM dataset [32]. This dataset is compiled from around 1000 published estimates of LAI covering the period from 1932 to 2000. These historical LAI data include natural and seminatural (managed) ecosystems and some cultivated vegetation. It contains a wide range of LAI values of 15 biome/land cover classes, from 0.46–2.16 for deserts to 4.4–13.04 for tree plantations. We downloaded it from the website (https://daac.ornl.gov/cgi-bin/dsviewer.pl?ds_id=584, accessed on 11 March 2021).

We extracted the Landsat-5 Tier 1 surface reflectance product for each LAI pixel using the LAI measurement date and geographical information (latitude and longitude). Then, we calculated VIs from these reflectance values. The specific steps are as follows:

(1) Data cleaning: data with null values for latitude, longitude, or date of measured LAI were removed. Also, data with LAI as a range value instead of a specific value were removed; data with LAI measurements spanning over one month were removed.
(2) Screening of data corresponding to dense vegetation: data with LAI values less than 4.0 were removed.
(3) Screening of time range: data with a LAI measurement date range outside the Landsat-5 coverage time range were removed.
(4) Removing duplicate data: some data had the same measurement time and geographical location with different LAI values. On closer examination, we found that some of these data were from measurements of different biomes. Therefore, these data were also removed as we could not establish a one-to-one relationship between LAI and VIs.
(5) Matching LAI with satellite reflectance image: because the temporal accuracy of all measured LAI data is only one month, we searched all surface reflectance data for the month of Landsat-5 Tier 1 data and averaged them to obtain the final surface reflectance data. Some reflectance data could not be retrieved due to cloud cover, so their corresponding LAI data were removed.

Table 1 shows the LAI data and corresponding station information that remained after the above steps. These data were used for evaluating the LAI estimation accuracy of VIs. The LAI value ranges from 4.06 to 10.59, and the sites are mainly located in Canada, Japan, and the USA.

### 2.2. Methods

#### 2.2.1. Vegetation Indices

Four VIs, including NDVI, SAVI, TSAVI, and EVI, were studied. The NDVI is the most commonly used VI. It is defined as:

(1) $$\mathrm{NDVI}=\frac{N-R}{N+R}$$

where *R* and *N* are the atmospherically corrected surface reflectance in the red and NIR bands.

Conversely to NDVI, SAVI and TSAVI have a soil adjustment factor designed to mitigate the soil noise by considering multiple soil scattering. Here, this factor is referred to as “*X*” with the VIs name as a suffix. SAVI is defined as:

(2) $$\mathrm{SAVI}=\frac{\left(N-R\right)(1+X_{\mathrm{SAVI}})}{N+R+X_{\mathrm{SAVI}}}$$

where *X*_SAVI_ is the soil adjustment factor of SAVI. A value equal to 0.5 is recommended in its original paper [11]. 

TSAVI is defined as:

(3) $$\mathrm{TSAVI}=\frac{a\cdot \left(N-a\cdot R-b\right)}{a\cdot N+R-a\cdot b+X_{\mathrm{TSAVI}}\cdot \left(1+a^{2}\right)}$$

where *a* and *b* are the slope and interception of the soil line, respectively. Here these two parameters were set as the constant value of 1.2 and 0.04, respectively, which are considered global soil line parameters [12]. *X*_TSAVI_ is the soil adjustment factor of TSAVI, recommended to be equal to 0.08 in the original paper [12].

EVI, in addition to a soil adjustment factor, uses atmosphere resistance factors:

(4) $$\mathrm{EVI}=G\frac{N-R}{N+C_{1}R-C_{2}B+X_{\mathrm{EVI}}}$$

where *B* is the atmospherically corrected surface reflectance in the blue band. *G* is a factor gain equal to 2.5. *C*_1_, *C*_2_ are the aerosol resistance coefficient (*C*_1_ *=* 6, *C*_2_ *=* 7.5), using the blue band reflectance to correct aerosol influences in the red band. *X*_EVI_ is a soil adjustment factor initially equal to 1 [19]. Since the surface reflectance products were used as multispectral images, we assumed that atmospheric influences had been almost completely removed. Therefore, atmosphere resistance was not considered in this paper.

#### 2.2.2. Time Series Analysis

Time series analysis is a statistical method that processes time series data to forecast, control, and understand features of the data. [33]. Time series of LAI can be separated into the trend, seasonality, and residuals. Trend is the increasing (or decreasing) value in the series data. Seasonality is the repeating short-term cycle in the data series. Residuals are the time series data after the trend, and seasonal components are removed. The decomposition was achieved using classical seasonal decomposition by moving averages [34]. The seasonal multiplication component was selected, and the base-level static analysis was conducted with the rolling mean and Dickey-Fuller test. As the threshold for this analysis, a 99% significance level was employed for the null hypothesis (i.e., no trend). This test explored the trend from a quarter of a year to the next. In the time series analysis, the independent variable was date, and the dependent variable was VIs or LAI. Considering that the field measured LAI data were not continuously observed, only MODIS LAI data were used for time series analysis.

#### 2.2.3. Linear Regression

Linear regression is a method to build the linear relationship between a dependent variable and one or more independent variables [35]. We used a unitary linear regression with VI as the independent variable and LAI as the dependent variable. The linear regression equation is defined as:

(5) LAI=c⋅VIs+d

where *c* is the slope and *d* is the interception of the regression line. The linear regress is widely used to estimate LAI from VIs for its simplicity [8,36]. When LAI increases to a certain threshold value and no longer maintains a linear relationship with VIs, the saturation effect is observed.

#### 2.2.4. Exhaustive Search

For discrete problems where no effective solution method is known, it may be necessary to test every possibility sequentially to determine whether it is the solution. Such exhaustive examination of all possibilities is called exhaustive search, direct search, or the “brute force” method [37]. The exhaustive search was used to evaluate the performance of SAVI calculated with exhaustive optimal soil adjustment factor *X*_SAVI_. Here the independent variable was *X*_SAVI_, and the dependent variable was *R*^2^, slope, and *P* of the linear regression equation, respectively. The work of Ren et al. [38] demonstrated that a negative *X*_SAVI_ value (e.g., −0.2) is acceptable, which allowed us to set the exhaustive interval of *X*_SAVI_ as [−0.3, 1] and to divide the boundary into 2000 parts at intervals of 0.001. The ability to estimate LAI using SAVI was investigated using a *X*_SAVI_ sequence over the whole study period. The estimation was achieved using a linear regression model between LAI and SAVI calculated with the *X*_SAVI_ sequence. Both MODIS and field measured LAI data were used here. Finally, the *R*^2^ and *P* were used to evaluate the linear regression performance of various SAVI values calculated with positive or negative *X*_SAVI_.

## 3. Time Series Analysis

### 3.1. Seasonal Decomposition of LAI

**Figure 2:**
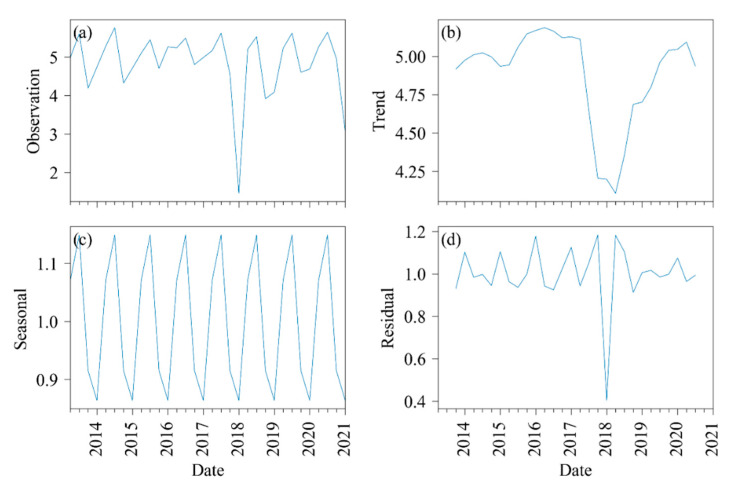
Decomposition analysis for the long-term LAI in the Apiacás (2013–2020). (**a**) LAI is decomposed into (**b**) trend, (**c**) seasonal, and (**d**) residual. The full-year LAI is separated into four quarters. Then, the mean value of each quarter is calculated and is used for time series analysis.

Time series analysis of LAI was conducted to ensure the stability of vegetation conditions in the study area. The result of the decomposition analysis of LAI time series can be seen in Figure 2. All LAI data (labeled as observation in the figure) were averaged using a quarter (i.e., winter, spring, summer, autumn). However, due to cloud cover, few quarters had no corresponding LAI. In that case, a mean value between the preceding and the following values of this null value was assigned. All quarterly LAI data were then decomposed into three parts: trend, seasonal, and residual with a quarterly frequency. 

**Figure 3:**
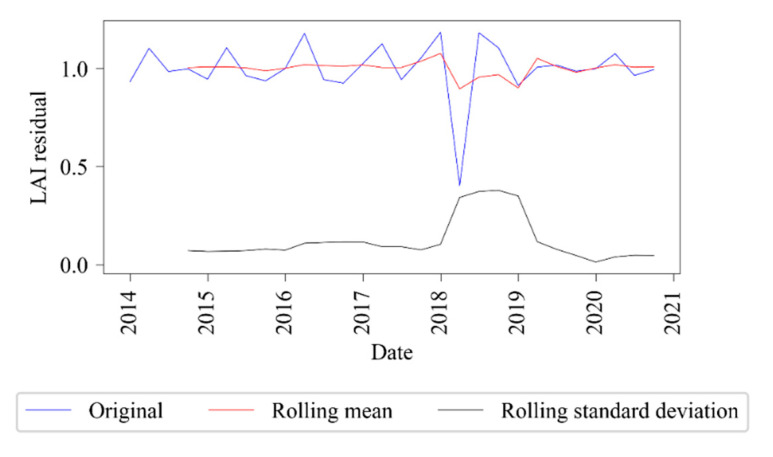
Stationarity analysis of MODIS LAI residual by rolling mean and standard deviation over the whole observation period.

The residual LAI stationarity was validated using the rolling mean (Figure 3) and the Dickey-Fuller test (Table 2). As shown in Figure 3, the means and variances of LAI tended to be constant with little volatility. According to the Dickey-Fuller Test results (Table 2), the statistic value was much smaller than the critical value at 1%, meaning that the data can be considered stable at the 99% confidence level.

### 3.2. Time Series Analysis of LAI and VIs

**Figure 4:**
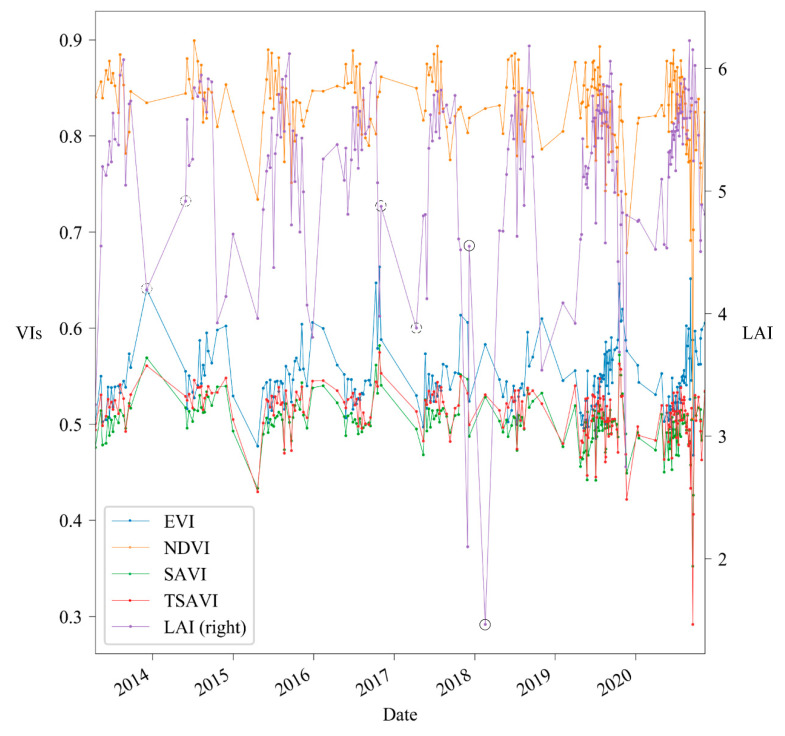
Time series of VIs (left axis) and LAI (right axis). The solid circles highlight a period when the LAI has a trend opposite to that of all VIs. The slash circles highlight a period when the trend of LAI is consistent with NDVI while being opposite to other VIs. The dashed circles highlight a period when the trend of LAI is consistent with all VIs.

The variations of VIs and LAI time series are plotted in Figure 4. It allows one to compare the performance of VIs on LAI estimation. The VIs were calculated with the default soil adjustment factor (*X*_SAVI_ = 0.5, *X*_TSAVI_ = 0.08, and *X*_EVI_ = 1). LAI values ranged from 2 to 6.5. NDVI had the highest value over the entire period (i.e., values from 0.7 to 0.9), followed by EVI (i.e., values from 0.4 to 0.7). SAVI and TSAVI shared almost the same range, from 0.2 to 0.6. These high values suggest that the area has high foliage coverage. Besides, Figure 4 shows that VIs can be indicators or not of LAI’s trends depending on time. The solid circles highlight a period when LAI has a trend opposite to that of all VIs. The slash circles highlight a period when the trend of LAI is consistent with NDVI while being opposite to other VIs. The dashed circles highlight a period when the trend of LAI is consistent with all VIs.

## 4. Optimal Soil Adjustment Factor *X*

### 4.1. Linear Regression between VIs and LAI

**Figure 5:**
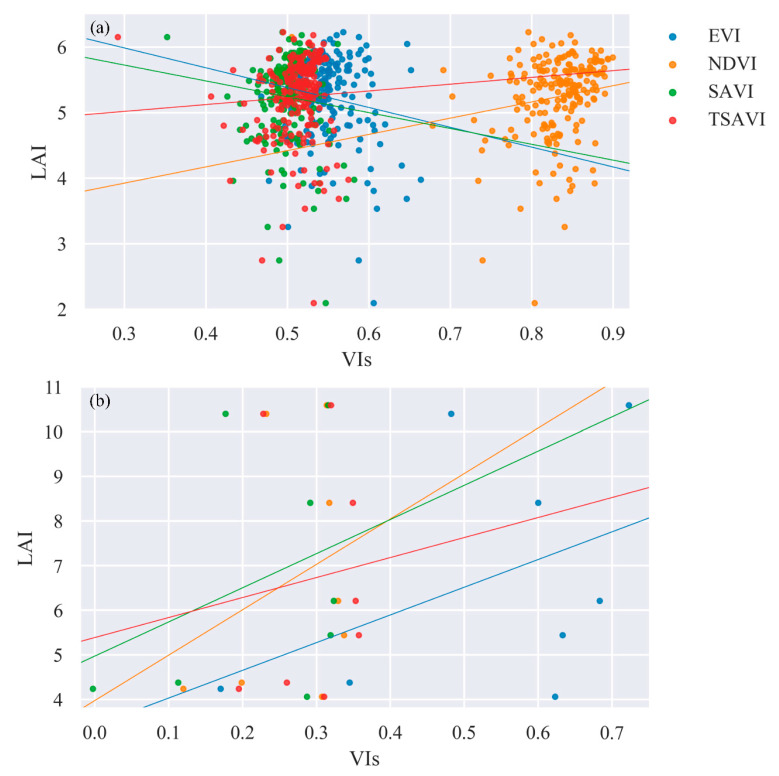
Scatter plot between VIs and (**a**) MODIS and (**b**) field measured LAI. The solid lines represent the trendlines of the linear regression model.

We tested the ability of VIs for LAI estimation. Figure 5 shows the scatter plot of LAI (y-axis) and VIs (x-axis). The linear regression model was used to compare the VIs. Table 3 gives the coefficients of the linear regression models. Figure 5 and Table 3 clearly show strong saturation effects for all VIs. The VIs hardly showed an upward trend with the increase of LAI. Besides, all VIs showed a weak correlation relationship with LAI. In general, NDVI showed relatively good performance in both cases, with the highest *R*^2^ (0.1632 in MODIS LAI and 0.4313 in field measured LAI) and the lowest *P* (0.0173 in MODIS LAI and 0.2860 in field measured LAI), followed by TSAVI. EVI and SAVI performances were unexpected, especially with the MODIS LAI data, with which they were negatively correlated. In all, the use of VIs to estimate LAI in very densely vegetated areas could lead to large errors.

### 4.2. LAI Estimation Using SAVI with Negative Soil Adjustment Factor X_SAVI_

**Figure 6:**
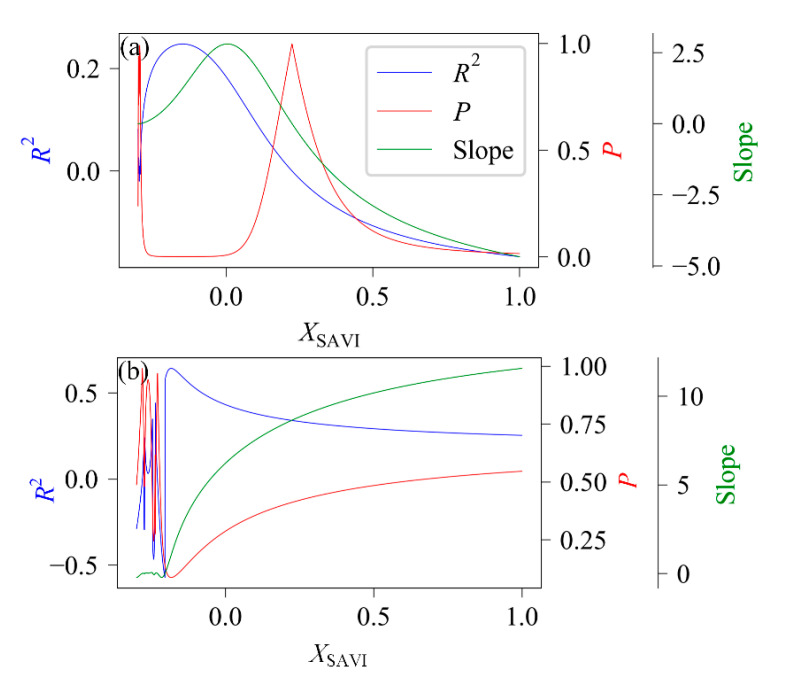
Linear regression between SAVI and (**a**) MODIS and (**b**) field-measured LAI with varying *X*_SAVI_. The optimal results are observed in the negative *X*_SAVI_ region.

We also conducted a linear regression analysis of SAVI and LAI by varying *X*_SAVI_ from −0.3 to 1 with a step of 0.001 to validate SAVI’s robustness using a negative soil adjustment factor. Both MODIS and field measured LAI data were used for the validation. The results of the linear regression are shown in Figure 6. 

From both LAI data, we can observe that the best results mainly appeared for *X*_SAVI_ between −0.18 and 0, indicating that negative *X*_SAVI_ values were very satisfactory and behaved well compared to positive values. In the MODIS LAI data (Figure 6a), the negative *R*^2^ and slope appeared in the interval from 0.225 to 1 for *X*_SAVI_, indicating that the correlation between SAVI and LAI was extremely weak. Besides, the negative slope meant a negative relationship between LAI and SAVI. The maximin *R*^2^ (0.2472) and minimum *P* (0.0003) appeared at *X* = −0.148. The negative *R*^2^ and slope in the field measured data (Figure 6b) appeared for *X*_SAVI_ between −0.3 and −0.2. Besides, *R*^2^ and *P* were found to be highly volatile in this region. The maximin *R*^2^ (0.6417) and minimum *P* (0.0863) appeared at *X*_SAVI_ = −0.183. With *X*_SAVI_ from −0.184 to 1, the performance of SAVI gradually worsened, with a decreasing *R*^2^ and an increasing *P*.

## 5. Discussion and Conclusions

### 5.1. Discussion

#### 5.1.1. Application Condition of the Negative Soil Adjustment Factor

According to a earlier study [11], the optimal value of the soil adjustment factor is related to the general condition of the vegetation, with *X*_SAVI_ close but smaller than one for sparse vegetation and close to but not smaller than 0 for dense vegetation. However, the lower boundary zero for dense vegetation was questioned by Ren et al. [38] based on field-measured data in arid grasslands and by Zhen et al. [8,14] based on simulated data by the discrete anisotropic radiative transfer [39,40,41,42,43,44,45,46,47,48] (DART, https://dart.omp.eu/#/, accessed on 11 March 2021) model. Remote sensing models such as DART that simulate the reflectance of land surfaces are very useful tools to investigate the domain of validity of VIs. More generally, they have a great potential to invert satellite observations in terms of LAI maps.

Similar to Ren et al. [38], we found an optimal performance of the negative soil adjustment factor. However, the vegetation conditions differed from the previous study [38] where arid grasslands gave had a NDVI of around 0.2, indicating very sparse vegetation. In our study area, the NDVI values were mainly distributed around 0.8, indicating very dense vegetation. We assumed that the better performance of negative soil adjustment factors in the two situations is because the intersections between the soil line and vegetation isolines were located in the first quadrant, even though their vegetation density varied dramatically.

This paper further confirmed that the lower boundary of the soil adjustment factor could be negative, based on field-measured and remote sensing data (Figure 6). Also, as was already pointed out by Zhen et al. [8] based on simulated data, the determining factors of the location of intersections between the soil line and vegetation isolines are determined by the interception of the vegetation isolines and the soil lines. When the vegetation isoline intercept is larger than the soil line intercept, the intersection point is located in the second or third quadrant of the red-NIR plane. When the vegetation isoline intercept is smaller than the soil line intercept, the intersection point is located in the first quadrant. Moreover, the intercept depends also largely on the spectral properties of the leaves and soil apart from canopy structure parameters such as LAI. Our study further confirmed this conclusion using both field-measured and remote sensing data.

Besides, we noticed that NDVI had a better performance than SAVI, which is considered an improved version of NDVI. Similar undesired SAVI performances have also been reported by [38,49]. Reasons for this poor performance may contribute to the fact that NDVI assumes vegetation isolines converge to the original point, and SAVI assumes vegetation isolines converge to one point (−0.5, −0.5) located in the third quadrant. In our study (very dense vegetation areas), the vegetation isolines converged to one point in the first quadrant. As a result, the assumption based on the original point is much better than the point (−0.5, −0.5). This reason can also be seen in the TSAVI slightly better performance than SAVI because TSAVI assumes that the vegetation isolines’ intersection lies between the origin and the point (−0.5, −0.5). Another possibility is that NDVI was used in the backup algorithm for inversion in MODIS LAI data when the radiative transfer inversion method fails, which might also influence the results.

#### 5.1.2. Saturation Effect

Numerous studies [8,11,12,13,15,16] have indicated that VIs show significant saturation effects with LAI growth. Several methods have been implemented to enhance the saturation resistance by adding spectral information or geometry information [19,36,50]. For example, EVI added a blue band which is more sensitive than the red band for high LAI [19]. The absorption intensity of the leaf in the blue band is less than that in the red band. When the LAI exceeded the threshold value, the canopy reflectance had almost no change in the red band due to the strong absorption intensity, and the canopy reflectance still decreased with the LAI because of the weaker absorption intensity in the blue band. Also, modified normalized difference vegetation index (MNDVI) [50] involved a combination of narrow bands in the shorter wavelengths of the red edge (700–750 nm) and longer wavelengths of the red edge (750–780 nm). Besides, NHVI [36] added an angular index representing the distribution of leaves inside the canopy to enhance the saturation resistance ability.

However, the increased information required leads to higher demands on the sensors. For example, EVI products cannot be produced from a sensor without the blue band (i.e., the advanced very high resolution radiometer, AVHRR). The narrowband requirement makes MNDVI hard to be calculated from some common sensors (i.e., Landsat 8). NHVI requires the sensor can capture multiple-angler observation. Therefore, it is a challenge for these VIs to produce long-term products across sensor systems with variable spectral response functions, swath width, and orbiting geometry. Compared with these methods, adjusting the soil adjustment factor may be effortless because it requires less information from the sensors. However, although the method using negative soil adjustment factor achieves better advantages in LAI estimation than other VIs, the accuracy is still lacking compared to these LAI estimation methods. Our method is, therefore, more suitable for use in situations where sensor conditions are limited. For example, when only an optic sensor with a fixed viewing direction is available.

In addition, we found that EVI and SAVI were negatively correlated with LAI in the MODIS LAI dataset. This negative correlation was not observed in the field measured data. It leads to inaccurate LAI estimation with errors that cannot be easily estimated. The negative correlation between VIs and the LAI is few reported in other VIs saturation studies, and we did not find a solid explanation for it. We simply assume that it is related to the insensitivity of reflectance values in the red band in the high LAI region. It will need to be further investigated in the future.

### 5.2. Conclusions

The saturation effects limit the use of VIs. In this study, we explored the possibility of using the negative soil adjustment factor to mitigate the saturation effects. Two data sets were used for the validation, including a long time series observation of MODIS LAI data in the Apiacás area and a global LAI dataset. The surface reflectance data derived from Sentinel-2, Landsat-8, and Landsat-5 were used for VIs calculation. An exhaustive search was employed to estimate the optimal soil adjustment factor for LAI estimation.

Results show that LAI was highly cyclical with seasonal changes. Besides, VIs calculated using the positive soil adjustment factor had serious saturation effects, and the correlation between VIs and LAI was very low. In the MODIS LAI data, SAVI and EVI even showed a negative growth with LAI. The exhaustive search suggested that a negative *X*_SAVI_ (e.g., −0.148) in MODIS LAI and *X*_SAVI_ (e.g., −0.183) in the field measured dataset might be better than the commonly used *X*_SAVI_ (e.g., 0.5) in a high LAI area.

So far, apart from the arid grasslands and simulated data that have been mentioned, the feasibility of using negative soil adjustment factors, our study indicates the feasibility of using a negative soil adjustment factor for saturation resistance in dense vegetation areas. Our study further confirms that the lower bound of the soil adjustment factor can be less than zero and reach negative regions. However, although the accuracy of using negative soil conditioning factors is much higher than positive values, the accuracy of our estimates still falls short compared to some other methods that are more demanding on the sensor. Therefore, the method proposed in this paper is more suitable for use with common optical satellites with single fixed observation direction.

## Figures and Tables

**Table 1:** Field measured LAI data from GLAIFM for evaluating the LAI estimation accuracy of VIs.

Site Name	Latitude (°)	Longitude (°)	LAI	Date
BOREAS NSA/OJP, Thompson	55.92	−98.62	4.38	July 1994
BOREAS NSA/OBS, Thompson	55.91	−98.45	4.06	July 1994
BOREAS NSA, Thompson	55.91	−98.52	8.41	July 1994
BOREAS NSA, Thompson	55.80	−98.00	6.21	July 1994
BOREAS NSA, Thompson	55.75	−97.80	5.44	July 1994
BOREAS SSA, Prince Albert	54.06	−105.93	10.59	August 1994
Arakawa River, Urawa	35.83	139.62	4.24	September 1985
Westvaco, Summerville, SC	33.20	−80.25	10.4	February 1991

**Table 2:** Results of Dickey-Fuller Test.

Parameters	Value
Test Statistic	−6.979
MacKinnon’s approximate *p*-value	8.281 × 10^−10^
Lags Used	1
Number of Observations Used	26
Critical value (1%)	−3.711
Critical value (5%)	−2.981
Critical value (10%)	−2.630

**Table 3:** Linear regression (slope, interception, *R*^2^, *P*) of the four VIs and LAI.

VIs	LAI Type	Slope	Interception	*R* ^2^	*P* *
NDVI	MODIS LAI	2.4769	3.1819	0.1632	0.0173
	Field measured LAI	6.2097	3.4104	0.4313	0.2860
SAVI	MODIS LAI	−2.4165	6.4499	−0.0904	0.1894
	Field measured LAI	10.1760	3.9748	0.2915	0.4836
TSAVI	MODIS LAI	1.0364	4.7082	0.0454	0.5103
	Field measured LAI	7.6553	4.9714	0.3386	0.4120
EVI	MODIS LAI	−3.0249	6.8936	−0.1504	0.0285
	Field measured LAI	4.4827	5.3874	0.1017	0.8106

* Two-sided *P* for a hypothesis test whose null hypothesis is that the slope is zero, using Wald Test with t-distribution of the test statistic.

## Data Availability

Landsat data are available at https://developers.google.com/earth-engine/datasets/catalog/landsat, accessed on 11 March 2021. Sentinel-2 data are available at https://developers.google.com/earth-engine/datasets/catalog/COPERNICUS_S2_SR#description, accessed on 11 March 2021. MODIS leaf area index 4-day global 500 m (MCD15A3H V6) are available at https://doi.org/10.5067/MODIS/MCD12Q1.006, accessed on 11 March 2021. The global leaf area index from field measurements, 1932–2000 data are available at https://daac.ornl.gov/cgi-bin/dsviewer.pl?ds_id=584, accessed on 11 March 2021.
